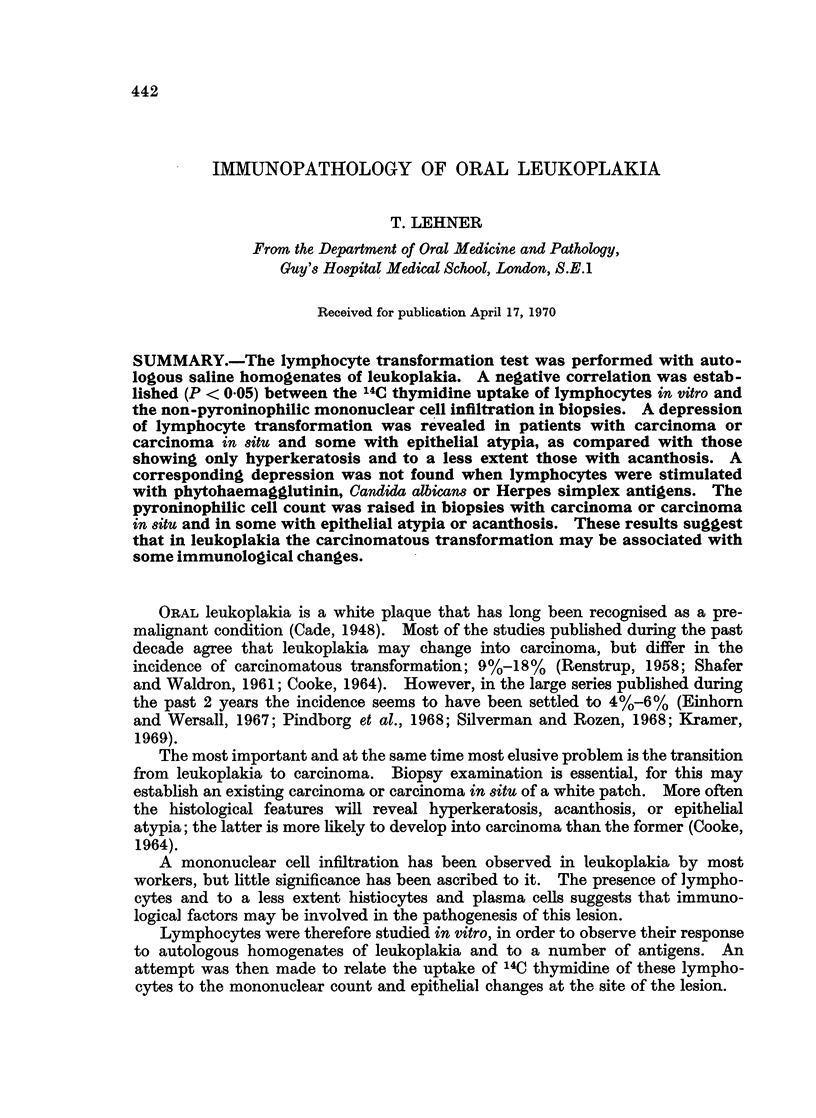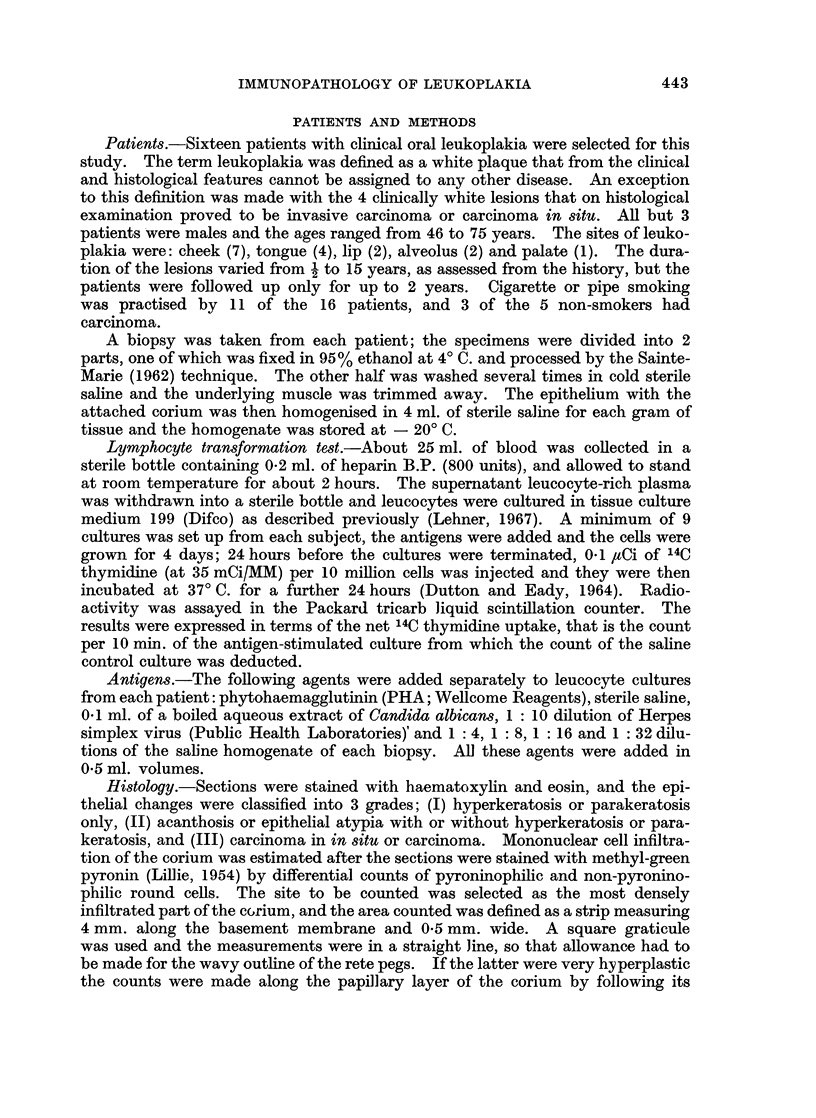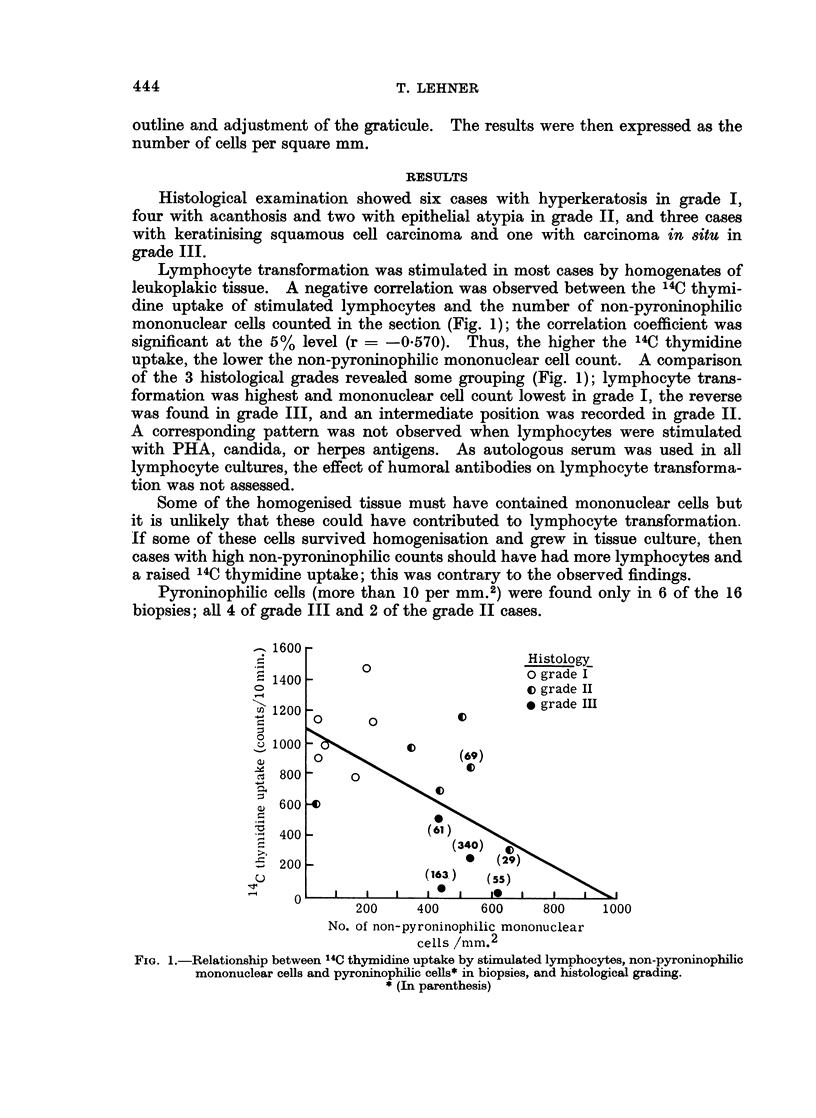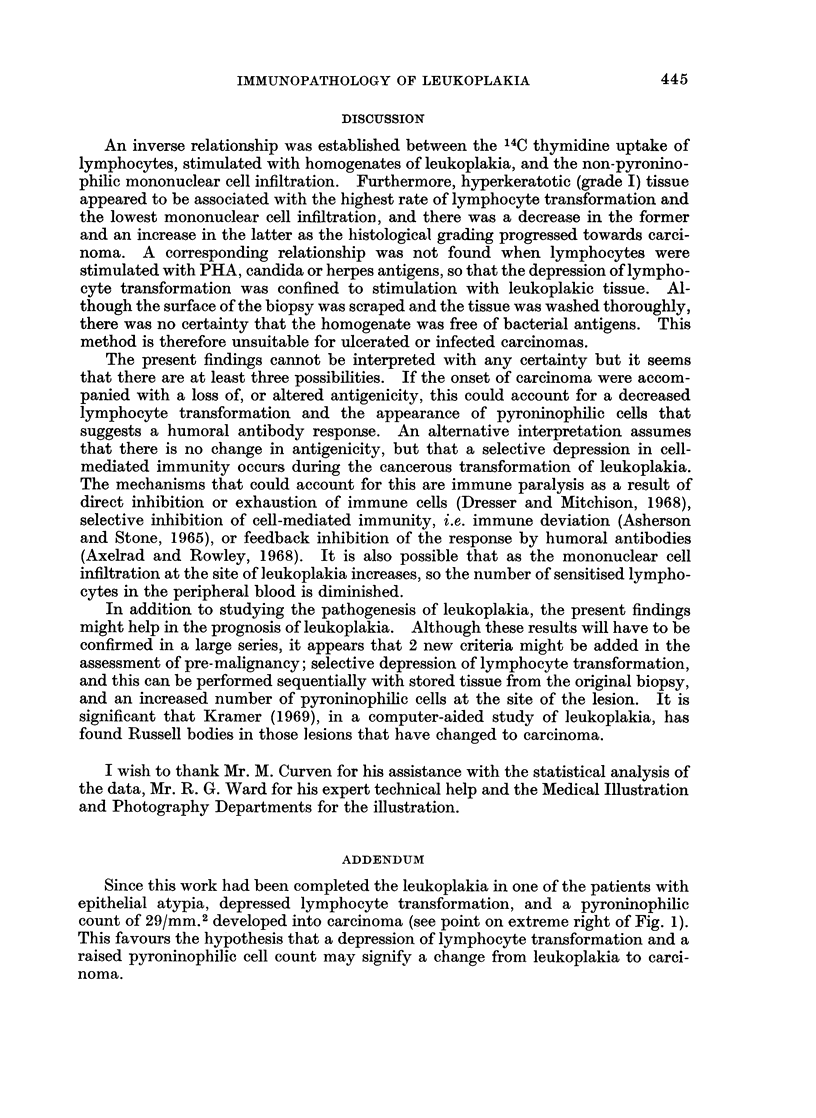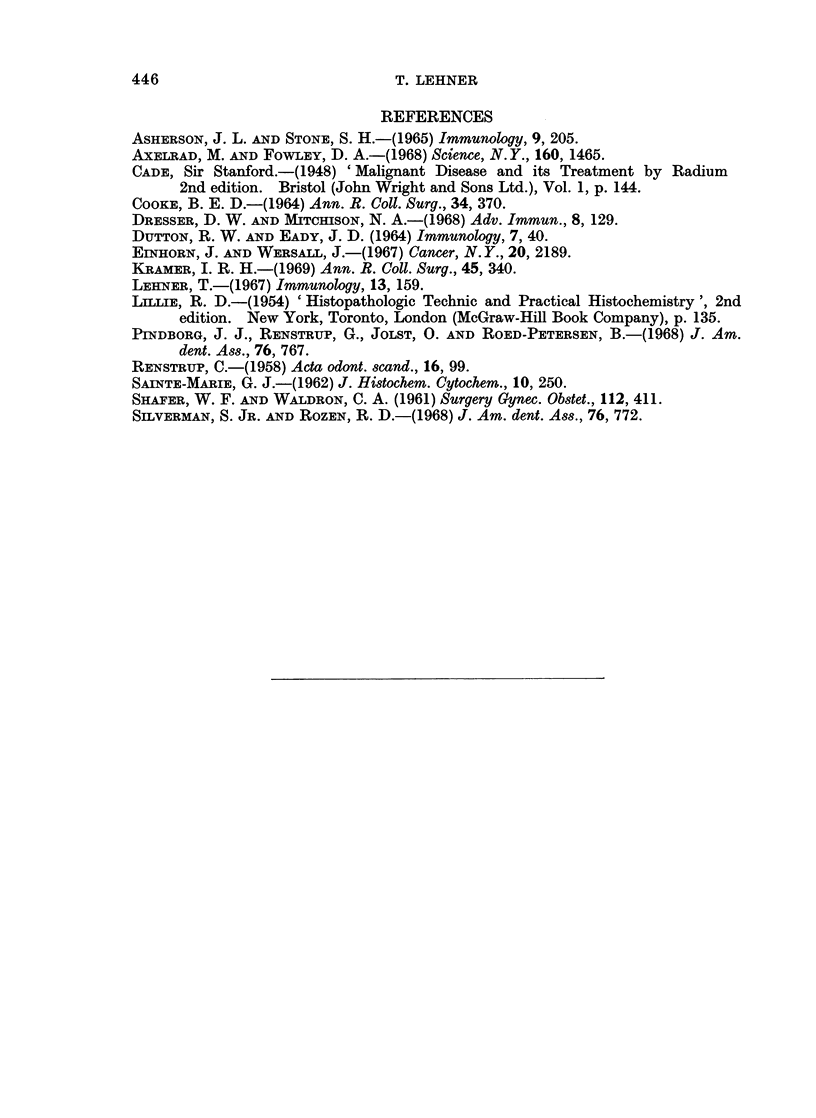# Immunopathology of Oral Leukoplakia

**DOI:** 10.1038/bjc.1970.52

**Published:** 1970-09

**Authors:** T. Lehner

## Abstract

The lymphocyte transformation test was performed with autologous saline homogenates of leukoplakia. A negative correlation was established (P<0.05) between the ^14^C thymidine uptake of lymphocytes *in vitro* and the non-pyroninophilic mononuclear cell infiltration in biopsies. A depression of lymphocyte transformation was revealed in patients with carcinoma or carcinoma *in situ* and some with epithelial atypia, as compared with those showing only hyperkeratosis and to a less extent those with acanthosis. A corresponding depression was not found when lymphocytes were stimulated with phytohaemagglutinin, *Candida albicans* or Herpes simplex antigens. The Pyroninophilic cell count was raised in biopsies with carcinoma or carcinoma *in situ* and in some with epithelial atypia or acanthosis. These result suggest that in leukoplakia the carcinomatous transformation may be associated with some immunological changes.


### IMMUNOPATHOLOGY OF ORAL LEUKOPLAKIA

T. LEHNER

*From the Department of Oral Medicine and Pathology, Guy's Hospital Medical School, London, S.E.*1

Received for publication April 17, 1970

**SUMMARY.—The lymphocyte transformation test was performed with autologous saline homogenates of leukoplakia. A negative correlation was established ($P < 0{\cdot}05$) between the $^{14}C$ thymidine uptake of lymphocytes *in vitro* and the non-pyroninophilic mononuclear cell infiltration in biopsies. A depression of lymphocyte transformation was revealed in patients with carcinoma or carcinoma *in situ* and some with epithelial atypia, as compared with those showing only hyperkeratosis and to a less extent those with acanthosis. A corresponding depression was not found when lymphocytes were stimulated with phytohaemagglutinin, *Candida albicans* or Herpes simplex antigens. The pyroninophilic cell count was raised in biopsies with carcinoma or carcinoma *in situ* and in some with epithelial atypia or acanthosis. These results suggest that in leukoplakia the carcinomatous transformation may be associated with some immunological changes.**

ORAL leukoplakia is a white plaque that has long been recognised as a premalignant condition (Cade, 1948). Most of the studies published during the past decade agree that leukoplakia may change into carcinoma, but differ in the incidence of carcinomatous transformation; 9%–18% (Renstrup, 1958; Shafer and Waldron, 1961; Cooke, 1964). However, in the large series published during the past 2 years the incidence seems to have been settled to 4%–6% (Einhorn and Wersall, 1967; Pindborg *et al.*, 1968; Silverman and Rozen, 1968; Kramer, 1969).

The most important and at the same time most elusive problem is the transition from leukoplakia to carcinoma. Biopsy examination is essential, for this may establish an existing carcinoma or carcinoma *in situ* of a white patch. More often the histological features will reveal hyperkeratosis, acanthosis, or epithelial atypia; the latter is more likely to develop into carcinoma than the former (Cooke, 1964).

A mononuclear cell infiltration has been observed in leukoplakia by most workers, but little significance has been ascribed to it. The presence of lymphocytes and to a less extent histiocytes and plasma cells suggests that immunological factors may be involved in the pathogenesis of this lesion.

Lymphocytes were therefore studied *in vitro*, in order to observe their response to autologous homogenates of leukoplakia and to a number of antigens. An attempt was then made to relate the uptake of $^{14}C$ thymidine of these lymphocytes to the mononuclear count and epithelial changes at the site of the lesion.

### PATIENTS AND METHODS

*Patients.*—Sixteen patients with clinical oral leukoplakia were selected for this study. The term leukoplakia was defined as a white plaque that from the clinical and histological features cannot be assigned to any other disease. An exception to this definition was made with the 4 clinically white lesions that on histological examination proved to be invasive carcinoma or carcinoma *in situ*. All but 3 patients were males and the ages ranged from 46 to 75 years. The sites of leukoplakia were: cheek (7), tongue (4), lip (2), alveolus (2) and palate (1). The duration of the lesions varied from $\frac{1}{2}$ to 15 years, as assessed from the history, but the patients were followed up only for up to 2 years. Cigarette or pipe smoking was practised by 11 of the 16 patients, and 3 of the 5 non-smokers had carcinoma.

A biopsy was taken from each patient; the specimens were divided into 2 parts, one of which was fixed in 95% ethanol at 4° C. and processed by the Sainte-Marie (1962) technique. The other half was washed several times in cold sterile saline and the underlying muscle was trimmed away. The epithelium with the attached corium was then homogenised in 4 ml. of sterile saline for each gram of tissue and the homogenate was stored at — 20° C.

*Lymphocyte transformation test.*—About 25 ml. of blood was collected in a sterile bottle containing 0·2 ml. of heparin B.P. (800 units), and allowed to stand at room temperature for about 2 hours. The supernatant leucocyte-rich plasma was withdrawn into a sterile bottle and leucocytes were cultured in tissue culture medium 199 (Difco) as described previously (Lehner, 1967). A minimum of 9 cultures was set up from each subject, the antigens were added and the cells were grown for 4 days; 24 hours before the cultures were terminated, 0·1 $\mu$Ci of $^{14}$C thymidine (at 35 mCi/MM) per 10 million cells was injected and they were then incubated at 37° C. for a further 24 hours (Dutton and Eady, 1964). Radioactivity was assayed in the Packard tricarb liquid scintillation counter. The results were expressed in terms of the net $^{14}$C thymidine uptake, that is the count per 10 min. of the antigen-stimulated culture from which the count of the saline control culture was deducted.

*Antigens.*—The following agents were added separately to leucocyte cultures from each patient: phytohaemagglutinin (PHA; Wellcome Reagents), sterile saline, 0·1 ml. of a boiled aqueous extract of *Candida albicans*, 1 : 10 dilution of Herpes simplex virus (Public Health Laboratories)' and 1 : 4, 1 : 8, 1 : 16 and 1 : 32 dilutions of the saline homogenate of each biopsy. All these agents were added in 0·5 ml. volumes.

*Histology.*—Sections were stained with haematoxylin and eosin, and the epithelial changes were classified into 3 grades; (I) hyperkeratosis or parakeratosis only, (II) acanthosis or epithelial atypia with or without hyperkeratosis or parakeratosis, and (III) carcinoma in *in situ* or carcinoma. Mononuclear cell infiltration of the corium was estimated after the sections were stained with methyl-green pyronin (Lillie, 1954) by differential counts of pyroninophilic and non-pyroninophilic round cells. The site to be counted was selected as the most densely infiltrated part of the corium, and the area counted was defined as a strip measuring 4 mm. along the basement membrane and 0·5 mm. wide. A square graticule was used and the measurements were in a straight line, so that allowance had to be made for the wavy outline of the rete pegs. If the latter were very hyperplastic the counts were made along the papillary layer of the corium by following its outline and adjustment of the graticule. The results were then expressed as the number of cells per square mm.

### RESULTS

Histological examination showed six cases with hyperkeratosis in grade I, four with acanthosis and two with epithelial atypia in grade II, and three cases with keratinising squamous cell carcinoma and one with carcinoma *in situ* in grade III.

Lymphocyte transformation was stimulated in most cases by homogenates of leukoplakic tissue. A negative correlation was observed between the $^{14}C$ thymidine uptake of stimulated lymphocytes and the number of non-pyroninophilic mononuclear cells counted in the section (Fig. 1); the correlation coefficient was significant at the 5% level (r = —0·570). Thus, the higher the $^{14}C$ thymidine uptake, the lower the non-pyroninophilic mononuclear cell count. A comparison of the 3 histological grades revealed some grouping (Fig. 1); lymphocyte transformation was highest and mononuclear cell count lowest in grade I, the reverse was found in grade III, and an intermediate position was recorded in grade II. A corresponding pattern was not observed when lymphocytes were stimulated with PHA, candida, or herpes antigens. As autologous serum was used in all lymphocyte cultures, the effect of humoral antibodies on lymphocyte transformation was not assessed.

Some of the homogenised tissue must have contained mononuclear cells but it is unlikely that these could have contributed to lymphocyte transformation. If some of these cells survived homogenisation and grew in tissue culture, then cases with high non-pyroninophilic counts should have had more lymphocytes and a raised $^{14}C$ thymidine uptake; this was contrary to the observed findings.

Pyroninophilic cells (more than 10 per mm.$^2$) were found only in 6 of the 16 biopsies; all 4 of grade III and 2 of the grade II cases.

FIG. 1.—Relationship between $^{14}C$ thymidine uptake by stimulated lymphocytes, non-pyroninophilic mononuclear cells and pyroninophilic cells* in biopsies, and histological grading.
* (In parenthesis)

### DISCUSSION

An inverse relationship was established between the $^{14}C$ thymidine uptake of lymphocytes, stimulated with homogenates of leukoplakia, and the non-pyroninophilic mononuclear cell infiltration. Furthermore, hyperkeratotic (grade I) tissue appeared to be associated with the highest rate of lymphocyte transformation and the lowest mononuclear cell infiltration, and there was a decrease in the former and an increase in the latter as the histological grading progressed towards carcinoma. A corresponding relationship was not found when lymphocytes were stimulated with PHA, candida or herpes antigens, so that the depression of lymphocyte transformation was confined to stimulation with leukoplakic tissue. Although the surface of the biopsy was scraped and the tissue was washed thoroughly, there was no certainty that the homogenate was free of bacterial antigens. This method is therefore unsuitable for ulcerated or infected carcinomas.

The present findings cannot be interpreted with any certainty but it seems that there are at least three possibilities. If the onset of carcinoma were accompanied with a loss of, or altered antigenicity, this could account for a decreased lymphocyte transformation and the appearance of pyroninophilic cells that suggests a humoral antibody response. An alternative interpretation assumes that there is no change in antigenicity, but that a selective depression in cell-mediated immunity occurs during the cancerous transformation of leukoplakia. The mechanisms that could account for this are immune paralysis as a result of direct inhibition or exhaustion of immune cells (Dresser and Mitchison, 1968), selective inhibition of cell-mediated immunity, *i.e.* immune deviation (Asherson and Stone, 1965), or feedback inhibition of the response by humoral antibodies (Axelrad and Rowley, 1968). It is also possible that as the mononuclear cell infiltration at the site of leukoplakia increases, so the number of sensitised lymphocytes in the peripheral blood is diminished.

In addition to studying the pathogenesis of leukoplakia, the present findings might help in the prognosis of leukoplakia. Although these results will have to be confirmed in a large series, it appears that 2 new criteria might be added in the assessment of pre-malignancy; selective depression of lymphocyte transformation, and this can be performed sequentially with stored tissue from the original biopsy, and an increased number of pyroninophilic cells at the site of the lesion. It is significant that Kramer (1969), in a computer-aided study of leukoplakia, has found Russell bodies in those lesions that have changed to carcinoma.

I wish to thank Mr. M. Curven for his assistance with the statistical analysis of the data, Mr. R. G. Ward for his expert technical help and the Medical Illustration and Photography Departments for the illustration.

### ADDENDUM

Since this work had been completed the leukoplakia in one of the patients with epithelial atypia, depressed lymphocyte transformation, and a pyroninophilic count of 29/mm.$^2$ developed into carcinoma (see point on extreme right of Fig. 1). This favours the hypothesis that a depression of lymphocyte transformation and a raised pyroninophilic cell count may signify a change from leukoplakia to carcinoma.

### REFERENCES

ASHERSON, J. L. AND STONE, S. H.—(1965) *Immunology*, **9**, 205.
AXELRAD, M. AND FOWLEY, D. A.—(1968) *Science, N.Y.*, **160**, 1465.
CADE, Sir Stanford.—(1948) 'Malignant Disease and its Treatment by Radium 2nd edition. Bristol (John Wright and Sons Ltd.), Vol. 1, p. 144.
COOKE, B. E. D.—(1964) *Ann. R. Coll. Surg.*, **34**, 370.
DRESSER, D. W. AND MITCHISON, N. A.—(1968) *Adv. Immun.*, **8**, 129.
DUTTON, R. W. AND EADY, J. D. (1964) *Immunology*, **7**, 40.
EINHORN, J. AND WERSALL, J.—(1967) *Cancer, N.Y.*, **20**, 2189.
KRAMER, I. R. H.—(1969) *Ann. R. Coll. Surg.*, **45**, 340.
LEHNER, T.—(1967) *Immunology*, **13**, 159.
LILLIE, R. D.—(1954) 'Histopathologic Technic and Practical Histochemistry', 2nd edition. New York, Toronto, London (McGraw-Hill Book Company), p. 135.
PINDBORG, J. J., RENSTRUP, G., JOLST, O. AND ROED-PETERSEN, B.—(1968) *J. Am. dent. Ass.*, **76**, 767.
RENSTRUP, C.—(1958) *Acta odont. scand.*, **16**, 99.
SAINTE-MARIE, G. J.—(1962) *J. Histochem. Cytochem.*, **10**, 250.
SHAFER, W. F. AND WALDRON, C. A. (1961) *Surgery Gynec. Obstet.*, **112**, 411.
SILVERMAN, S. JR. AND ROZEN, R. D.—(1968) *J. Am. dent. Ass.*, **76**, 772.